# Lower autonomic arousal as a risk factor for criminal offending and unintentional injuries among female conscripts

**DOI:** 10.1371/journal.pone.0297639

**Published:** 2024-03-27

**Authors:** Sofi Oskarsson, Anneli Andersson, Bridget M. Bertoldi, Antti Latvala, Ralf Kuja-Halkola, Brittany Evans, Adrian Raine, Christopher J. Patrick, Henrik Larsson, Catherine Tuvblad

**Affiliations:** 1 School of Behavioural, Social and Legal Sciences, Örebro University, Örebro, Sweden; 2 Department of Psychology, Florida State University, Florida, FL, United States of America; 3 Institute of Criminology and Legal Policy, University of Helsinki, Helsinki, Finland; 4 Department of Medical Epidemiology and Biostatistics, Karolinska Institutet, Solna, Sweden; 5 Department of Criminology, Psychiatry, and Psychology, University of Pennsylvania, Philadelphia, PA, United States of America; 6 School of Medical Sciences, Örebro University, Örebro, Sweden; 7 Department of Psychology, University of Southern California, Los Angeles, CA, United States of America; Kore University of Enna: Universita degli Studi di Enna ’Kore’, ITALY

## Abstract

**Background:**

Lower autonomic arousal is a well-known correlate of criminal offending and other risk-taking behaviors in men, but few studies have investigated this association in women.

**Aim:**

To test associations between autonomic arousal and criminal offending as well as unintentional injuries among female conscripts.

**Methods:**

All women born 1958–1994 in Sweden who participated in voluntary military conscription (n = 12,499) were identified by linking Swedish population-based registers. Predictors were resting heart rate (RHR) and systolic blood pressure (SBP). Covariates were height, weight, and physical energy capacity. Main outcomes were criminal convictions (any, violent, and non-violent) from the National Crime Register. Secondary outcome was unintentional injuries requiring medical treatment or causing death. We used survival analyses to test for associations between predictors and outcomes.

**Results:**

Low RHR, relative to high RHR, was associated with an increased risk of any criminal conviction, non-violent criminal convictions, and unintentional injuries. Low SBP, relative to high SBP, was associated with an increased risk of violent criminal convictions.

**Conclusions:**

Results support lower autonomic arousal, particularly lower RHR, as a correlate of criminal offending among women that warrants further examination, as the reported findings have potential implications for the prediction of future female crime.

## Introduction

Criminal offending is an important public health issue as it poses a burden on victims, families, and offenders, as well as putting an economic strain on societies [1]. While the study of crime is extensive and spans numerous disciplines, preventative efforts targeting crime have been less successful compared to prevention programs for other societal outcomes [2]. Intervention and prevention efforts for crime typically focus on structural and social-level risk factors, but also individual-level risk factors such as personality traits and behaviors. However, to improve prediction and prevention strategies further, we may need to consider *additional* individual-level biological risk factors for crime. Attention has been given to the role of the autonomic nervous system, with a specific focus on resting heart rate (RHR) and to some extent also systolic blood pressure (SBP) as individual-level biological risk factors for criminal offending [3]. While the association between low RHR and criminal offending is well-established in males [3–6], little is known about this association in females.

### Autonomic arousal

RHR and SBP are both influenced by the sympathetic and the parasympathetic branches of the autonomic nervous system, with the parasympathetic branch being dominating under resting conditions [7]. Blood pressure is the force with which the blood moves through the vessels, while the heart rate is the number of times the heart beats each minute. Even though blood pressure and heart rate have a shared physiological base, heart rate and blood pressure do not always rise and fall in parallell, i.e., a rise in heart rate does not necessarily imply that the blood pressure increase at the same rate [7]. However, heart rate and blood pressure are often synchronized.

### Autonomic arousal and criminal offending

A substantial body of research has demonstrated that a lower RHR is associated with an elevated risk of criminal offending [4]. Yet in comparison, little attention has been paid to the potential association between SBP and criminal offending. The few studies that have investigated the relationship between SBP and criminal offending have all shown similar associations as RHR and crime, i.e., a lower SBP has been linked to an increased likelihood of criminal offending [8–11].

#### Theoretical explanations

While we do not know the precise mechanisms that underlie the association between autonomic nervous system functioning and criminal offending, two theories have dominated the literature thus far: 1) fearlessness theory and 2) stimulation-seeking theory. Fearlessness theory postulates that the situation during which RHR is measured is in fact mildly stressful and anxiety provoking given the novelty of the lab setting [12]. Some individuals will experience an increase in their heart rate during this time, while others will not. A low RHR is theorized to reflect fearlessness (i.e., attenuated reactivity to unfamiliar situations and aversive stimuli) which may increase the risk of sensation-seeking behaviors, which in theory includes criminal offending. Stimulation-seeking theory suggests that low autonomic arousal is an unpleasant physiological state which is relieved by engagement in risky behaviors (such as criminal offending), which increases the physiological arousal to more optimal levels [12, 13].

RHR and SBP have not only been linked to an elevated risk of criminal offending [9, 10] but earlier work has also shown that lower RHR and lower SBP are associated with an increased risk of being unintentionally injured [9]. It may be that the same theoretical perspectives that help us understand the association between lower autonomic arousal and criminal offending can also be applied to the understanding of the association between lower autonomic arousal and unintentional injury [14]. Individuals who are fearless and/or who exhibit an unpleasant under-arousal may be more likely to engage in risky situations and tend to disregard safety.

### Autonomic arousal and criminal offending among women

Women have in general a higher RHR than men [15], and they commit fewer crimes than men [16]. These facts have given rise to the question of whether lower RHR may be a potential explanation for the greater involvement of men in crime [14]. Most studies investigating the association between RHR and criminal offending have been conducted using male samples. Although a handful of studies reported associations of lower RHR with criminal offending in female samples as well [17–19] others have failed to support this association in women [20]. Five meta-analyses presented evidence for a relationship between RHR and antisocial behavior, which typically includes criminal offending: [6]—antisocial behavior: Cohen’s *d* = -.44; [5]—aggression: *d* = -.38, and conduct problems: *d* = -.33, [3]—antisocial behavior: *d* = -.20, [4]—antisocial behavior: Hedges *g* = -.17. The meta-analyses that examined various moderators did not find that sex moderated the association [3, 4, 6].

Even though results from previous studies suggest that RHR may be associated with criminal offending also in women, earlier studies including women have in general been limited by small and selected samples, typically utilizing cross-sectional designs. Based on the information available in the foregoing meta-analyses, the largest female samples in studies examining the association between RHR and criminal offending included a few thousand participants at most (e.g., 2,256 women [18]). Large-scale studies employing long-term follow-up evaluations to test for associations of both RHR and SBP with criminal offending in women do not, to the authors’ knowledge, exist at this time. Thus, while the association between autonomic nervous system functioning and criminal offending has been firmly established in men, the evidence is less convincing for women. Given the clear sex differences in both autonomic nervous system functioning [15] as well as criminal offending [21], it is necessary to comprehensively examine this association in women as has been done in men. Results from the current study may aid future research in considering potential predictors for criminal offending to be included in risk assessment tools targeted toward women.

## Objective

Given these knowledge gaps and the methodological limitations of previous studies, the overarching aim with the present study was to explore associations between measures of autonomic arousal with criminal offending and unintentional injuries in women. More specifically, the present study was undertaken to test for associations of RHR and SBP with criminal offending as well as unintentional injuries in a large-scale all-female conscript sample that was followed for up to 40 years. We sought to address among females in this sample whether 1) lower RHR was associated with an increased risk of criminal offending and/or unintentional injuries, and 2) lower SBP was associated with an increased risk of criminal offending and/or unintentional injuries.

## Material and methods

### Data sources

Several Swedish nationwide population-based registers were linked using the unique personal identity number (PIN) that has been assigned to all individuals living and residing in Sweden since 1947 [22]; this unique numeric identifier makes it possible to access data for the same individuals from several registers. The linkage of the registers was previously approved in written format by the Regional Ethical Review Board in Stockholm (2013/862-31/5) and data was accessed from November 1^st^ 2021 and onward. For the present study we used data from the following seven registers: (1) the *Total Population Register* (TPR), which was established in 1968 and provides demographic information such as age, sex, death, and emigration on all Swedish citizens [23]; (2) the *Swedish Military Conscription Register*, which contains information from a conscription assessment conducted to estimate each conscript’s ability to carry out military service and to determine the position within the military the conscript was best suited to [24]; (3) the *National Crime Register* (NCR), which contains information on all persons convicted of a criminal offense by a Swedish district court at or after age 15 (the minimum age of criminal responsibility in Sweden); (4) the *National Census Records*, which contains information on family socioeconomic status; (5) the *National Patient Register* (NPR), which started in 1964 and contains information about inpatient and outpatient psychiatric and somatic care; (6) the *Cause of Death Register* (CDR), containing information about individual causes of death; and (7) the *Longitudinal Integration Database for Health Insurance and Labour Market Studies* (LISA) which includes information about health, socioeconomic factors, and the labor market [25].

### Study population

We identified all women born in Sweden between the years 1958 and 1994 (*N* = 1,889,669) as women born within and outside of Sweden may differ in their chance of conscription. The included age span ensured that the oldest individuals were 15 years of age at the start of the NCR (i.e., in 1973) and that all individuals had at least one year to conscript following their 18^th^ birthday. We excluded those with an immigration status at any point in time (5% of the initial cohort, *n* = 105,191), and those who emigrated (1.8% of the initial cohort *n* = 34,520) or died (1.1% of the initial cohort, *n* = 20,753) before or on their 15^th^ birthday. From these women, we identified 15,053 (1% of the initial cohort) who had been conscripted. More detailed information about female conscription in Sweden is provided below. We excluded those who emigrated before conscription (*n* = 1) or could not be linked to their biological mother (*n* = 8), and those who had missing values for both RHR and SBP (17% of the conscripted cohort *n* = 2,545). The final sample consisted of 12,499 (83% of the conscripted cohort) female conscripts, with the youngest ones having been born in 1992.

### Predictors

#### Resting heart rate and systolic blood pressure

During the conscription assessment, conscripts underwent a physical examination in which RHR was obtained. Conscripts were lying down in the supine position and rested for 5–10 minutes before RHR and SBP were measured using an arm cuff positioned at heart level [26]. This is a standard procedure for obtaining RHR and SBP in clinical settings in Sweden. In line with previous studies, [9–11], no woman was excluded due to extreme values for RHR (lower than 35 beats per minute [bpm] or higher than 145 bpm) or SBP (lower than 80 millimeter of mercury [mmHg] or higher than 160 mmHg.

A value for RHR was available for more than 85% of the sample (n = 10,682). Reasons for missing RHR data have been reported elsewhere [9, 26]. A value for SBP was available for more than 99% of the sample (n = 12,491). In line with prior research [9–11], RHR and SBP were divided into quintiles, in line with previous research to account for the potential of non-linear relationships. In the present sample, RHR and SBP were weakly positively correlated, *r*(10672) = 0.26, p < .001.

### Outcomes

#### Criminal convictions

We used criminal convictions, categorized as follows, as the main outcome of interest in the present study; any criminal conviction, violent, or non-violent criminal conviction. In line with previous research, violent criminal convictions were defined as convictions for homicide, manslaughter, assault, kidnapping, illegal confinement, unlawful coercion, gross violation of a person’s or a woman’s integrity, unlawful threats, intimidation, robbery, arson, and threats and violence against an officer [11, 27]. Crimes other than these were defined as non-violent criminal convictions [11, 27]. It is important to note that the span of included criminal convictions range from minor offenses to felonies, for any, violent, and non-violent convictions. However, given the relatively small sample size and the rare nature of the outcome among women, we were unable to conduct analyses stratified on severity.

#### Unintentional injury

Lower RHR and lower SBP have in earlier studies been associated with an increased risk of unintentional injuries in men, an association that has been hypothesized to be a potential result of fearless or risk-taking behavior [9]. Therefore, we used unintentional injuries as a secondary outcome. We defined unintentional injury in line with previous research [9], including inpatient or outpatient treatments and deaths that were due to unintentional injury (i.e., not self-harm, e.g., transportation crashes and falls). Unintentional injury was coded using the World Health Organization’s (WHO) International Classification of Disease (ICD; ICD-8 codes E800-E929; ICD-9 codes E800-E869; ICD-10 codes V01-X59).

### Covariates

Most of the measures included as covariates in the reported analyses were obtained during the military conscription assessment. Height and weight were included as they tend to be associated with both RHR and criminal offending [28]. Physical energy capacity, another correlate of RHR [29] and potentially also of ciminal offending [9], was also included as a covariate. Physical energy capacity was measured in Watts from a cycle ergometer during physical exercise, with a higher wattage level indicating a better physical performance [30]. Physical energy capacity was further divided by weight to account for body size [9]. Lastly, we adjusted for participant birth year to account for potential cohort or period effects.

### Statistical analyses

Data management and analyses were performed using version 9.4 of the SAS software package (SAS Institute Inc., Cary, NC) and R version 4.0.5 [31]. We tested for associations between RHR and SBP as the predictor variables, with criminal convictions and unintentional injuries as the outcome variables using survival analyses. Cox proportional hazards regression models were used to compute both crude and adjusted hazard ratios (HRs) as estimates of relative risk for each outcome at follow-up, together with 95% confidence intervals (CIs). We interpreted a HR greater than 1 as an increased risk of the outcomes, and a HR below 1 as a decreased risk of the outcome. The follow-up interval began from the date that the participants turned 15 years of age (minimum age of criminal responsibility in Sweden) and ended when participants either (1) experienced the outcome (i.e., date of conviction), (2) emigrated, (3) died, or (4) reached the end of the study period (i.e., December 31^st^, 2013). Hence, participants were followed for up to 40 years.

We conducted separate analyses for each of the three criminal offense outcomes (i.e., any, violent, and non-violent criminal convictions). For all analyses, we used age as the underlying time scale. All models accounted for the non-independence of siblings (sisters) by using a cluster-robust sandwich estimator, which takes into account the correlation between observations within clusters. We used Schoenfeld residuals to test the proportionality assumption. There was no violation in any of the covariates except birth year, which we addressed by stratification.

### Sensitivity analysis

To test the robustness of our results, we stratified our sample to include only female conscripts who had conscripted before their 21^st^ birthday to minimize potential effects of developmental changes. Further, in line with previous research [11], we allowed for the outcome to occur either before or after conscription (i.e., when RHR and SBP data was obtained) in our main analyses. We therefore stratified our sample to include only those who experienced the outcome (i.e., being convicted or unintentionally injured for the first time) after conscription. Unsurprisingly given that crime peaks in adolescence [32], this approach reduced the number of individuals with criminal convictions by more than half for all criminal conviction outcomes (i.e., any, violent, and non-violent). Thus, this limited the statistical power even further and we were unable to run analyses using violent criminal convictions as the outcome.

### Comparing female conscripts to female non-conscripts

Women have been able to conscript in Sweden since the 1970s [33]. Unlike for men, conscription has been optional which makes female conscripts a highly select group. Because of this, we compared the female conscripts to female non-conscripts on several key descriptive variables to find out if and how these two groups of women differ.

For this purpose, we utilized the initial sample described in the section titled “Study Population,” excluding women who had conscripted. This yielded a sample of 1,714,152 women (i.e., non-conscripts). We compared the two groups of women on the outcome variables: any, violent, and non-violent criminal convictions as well as unintentional injuries using Cox proportional hazards regression to estimate HRs with 95% CI. As with the conscript sample, the follow-up period for this non-conscript sample spanned the day participants turned 15 years of age through to when they either (1) experienced the outcome, (2) emigrated, (3) died, or (4) reached the end of the study period. This analytic approach permitted examination of whether female conscripts had a higher or lower risk of being convicted of a criminal offense or being unintentionally injured, compared to female non-conscripts. We also used the same approach to compare the two groups of women on whether they had ever been diagnosed with a psychiatric disorder, except we started following the participants at birth instead of on their 15^th^ birthday.

As a next step, we compared childhood socioeconomic status (SES), as defined by the occupation of the head of the household during childhood, coded as low, medium, or high [9, 11]. We also compared the two groups of women on their highest achieved education: coded as low (<9 years-11-years), medium (12 years-14 years), high (15 years or more). Since both childhood SES and highest achieved education were ordinal variables, we used the non-parametric statistical test Mann-Whitney U test to compare the two groups of women on each.

## Results

### Descriptive statistics

Baseline characteristics of the female conscripts are displayed in Table 1. Baseline characteristics of the female non-conscripts are presented in the Supporting Information (S1 Table). The Kaplan-Meier survival curves for RHR and SBP with criminal offending and unintentional injuries can be found in S1 and S2 Figs of the Supporting Information.

**Table 1:** Descriptive information for female conscripts.

Variables	No. (%) with data	M (SD)
Age at conscription, y	12,499 (100)	18.8 (1.6)
Age at first criminal conviction, y	952 (7.6)	20.2 (6.0)
Age at first violent criminal conviction, y	84 (0.7)	21.6 (6.1)
Age at first non-violent criminal conviction, y	910 (7.3)	20.2 (6.0)
Age at first unintentional injury, y	4,263 (34.1)	24.4 (7.6)
Resting heart rate, bpm	10,682 (85.5)	73.3 (12.5)
Systolic blood pressure, mmHg	12,491 (99.9)	123.7 (11.0)
Weight, kg	12,484 (99.9)	64.4 (9.0)
Height, cm	12,484 (99.9)	167.8 (6.0)
Physical energy capacity, W	10,557 (85.0)	235.5 (35.3)
**Any psychiatric disorder**		
Yes	1,947 (15.6)	
No	10,552 (84.4)	
**Childhood SES**		
Low	3,425 (27.4)	
Medium	4,056 (32.5)	
High	2,555 (20.4)	
Missing	2,463 (19.7)	
**Highest achieved education**		
Low	642 (5.1)	
Medium	7,330 (58.6)	
High	4,521 (36.2)	
Missing	7 (0.1)	

Abbreviations: y (years), bpm (beats per minute), mmHg (millimeter of mercury), kg (kilogram), cm (centimeter), W (Watt), SES (socioeconomic status).

Table 2 display cumulative incidences (i.e., estimated probability) of any. violent, and non-violent convictions by quintiles of RHR and SBP. As expected, the cumulative incidences for violent convictions were lower than for non-violent convictions.

**Table 2:** Cumulative incidence rates of any, violent, and non-violent convictions for female conscripts.

Variable	No. (%) with data	Number of outcomes			Cumulative incidence of first conviction in %		
		Any	Violent	Non-violent	Any	Violent	Non-violent
**Resting heart rate, bpm**	10,673 (85)						
1^st^ quintile (35–59)	2,093 (17)	157	16	147	.081	.008	.076
2^nd^ quintile (60–66)	2,136 (17)	158	17	149	.078	.008	.073
3^rd^ quintile (67–73)	2,070 (17)	157	12	153	.077	.007	.075
4^th^ quintile (74–81)	2,016 (16)	143	14	138	.071	.006	.070
5^th^ quintile (82–142)	2,358 (19)	150	15	138	.066	.007	.061
**Systolic blood pressure, mmHg**	12,491 (99)						
1^st^ quintile (80–119)	2,310 (18)	213	27	198	.097	.013	.090
2^nd^ quintile (120–125)	1,573 (13)	129	8	127	.081	.005	.080
3^rd^ quintile (126–131)	3,324 (27)	217	22	202	.065	.006	.061
4^th^ quintile (132–139)	2,717 (22)	188	14	183	.067	.005	.066
5^th^ quintile (140–160)	2,567 (21)	204	13	199	.078	.005	.075

### Associations between lower resting heart rate and criminal offending

Table 3 displays the results from the Cox proportional hazards regression models for RHR and criminal offending. Unadjusted estimates are presented in S2 Table of the Supporting Information. In fully adjusted models, we found that female conscripts with the lowest RHR (≤62 bpm) had an increased risk for any criminal conviction (HR = 1.35, 95% CI: 1.04, 1.76) compared to female conscripts with the highest RHR (≥83 bpm). Female conscripts in the second RHR quintile (63–69 bpm) had an increased risk of any criminal conviction, with similar estimates similar to those for individuals in the first quintile (HR = 1.36, 95% CI: 1.05, 1.76), in each case relative to female conscripts with the highest RHRs (≥83 bpm). Associations between RHR and any criminal offending among female conscripts in the third (70–75 bpm) and fourth RHR quintiles (76–82 bpm) were not significant, as indicated by CIs overlapping a value of 1.

**Table 3:** Cox proportional hazards regression models for resting heart rate with criminal offending.

	Hazard Ratio (95% CI)	
Quintiles for RHR in bpm	Adjusted HRs^a^	Adjusted HRs^b^
**All criminal convictions**		
1^st^ (38–62)	**1.35 (1.08–1.70)**	**1.35 (1.04–1.76)**
2^nd^ (63–69)	**1.28 (1.02–1.61)**	**1.36 (1.05–1.76)**
3^rd^ (70–75)	**1.29 (1.03–1.62)**	1.26 (0.98–1.64)
4^th^ (76–82)	1.16 (0.92–1.46)	1.16 (0.90–1.52)
5^th^ (83–145)	1^c^	1^c^
**Violent convictions**		
1^st^ (38–62)	1.34 (0.66–2.74)	1.72 (0.72–4.10)
2^nd^ (63–69)	1.35 (0.67–2.74)	2.12 (0.94–4.79)
3^rd^ (70–75)	0.98 (0.45–2.12)	1.43 (0.59–3.50)
4^th^ (76–82)	1.13 (0.54–2.35)	1.19 (0.48–2.98)
5^th^ (83–145)	1^c^	1^c^
**Non-violent convictions**		
1^st^ (38–62)	**1.39 (1.09–1.75)**	**1.36 (1.03–1.78)**
2^nd^ (63–69)	**1.32 (1.05–1.67)**	**1.37 (1.05–1.78)**
3^rd^ (70–75)	**1.37 (1.09–1.73)**	**1.32 (1.01–1.72)**
4^th^ (76–82)	1.22 (0.96–1.54)	1.20 (0.92–1.57)
5^th^ (83–145)	1^c^	1^c^

Abbreviations: RHR (resting heart rate) bpm (beats per minute).

^a^Adjusted for birth year

^b^Adjusted for birth year, physical energy capacity, height, and weight.

^c^Category of comparison.

Associations between RHR and violent criminal convictions were not significant for female conscripts in any of the four quintiles of RHR compared to the female conscripts in the fifth quintile. This is likely due to insufficient statistical power (0.7% of the cohort had a violent conviction), as evidenced by the wide CIs for the estimates. However, fully adjusted models showed that female conscripts with the lowest RHRs (≤62 bpm) had a higher risk of a non-violent criminal conviction (HR = 1.36, 95% CI: 1.03, 1.78) compared to female conscripts with the highest RHRs (≥83 bpm). Similar estimates were found for female conscripts in the second quintile (63–69 bpm), who had an increased risk of a non-violent criminal conviction (HR = 1.37, 95% CI: 1.05, 1.78) relative to female conscripts with the highest RHRs (≥83 bpm). Comparable estimates were seen also for female conscripts in the third quintile (70–75 bpm), who had an increased risk of a non-violent criminal conviction (HR = 1.32, 95% CI: 1.01, 1.72) compared to female conscripts with the highest RHRs (≥83 bpm). Associations between RHR and non-violent criminal convictions were not significant for female conscripts in the fourth RHR quintile.

While we found evidence of associations between RHR and any criminal conviction, violent criminal convictions (although not significant) and non-violent criminal convictions using Cox proportional hazard regressions, a test of all categories (i.e., RHR quintiles) being null simultaneously yielded a p-value = 0.12 for any criminal conviction, a p-value = 0.44 for violent criminal convictions, and a p-value = 0.12 for non-violent criminal convictions. While we cannot rule out that RHR is unrelated to criminal convictions, these findings likely reflect insufficient statistical power.

### Associations between lower systolic blood pressure and criminal offending

Table 4 displays the results from the Cox proportional hazards regression models for SBP and criminal offending. Unadjusted estimates can be found in S3 Table of the Supporting Information. In models adjusted for birth year, we found that female conscripts with the lowest SBPs (≤113 mmHg) had an increased risk for any criminal conviction (HR = 1.26, 95% CI: 1.04, 1.52) relative to female conscripts with the highest SBPs (≥134 mmHg). This result was attenuated toward the null in fully adjusted models. Associations between SBP and any criminal offending among female conscripts in the second (114–119 mmHg), third (120–125 mmHg) and fourth SBP quintiles (126–133 mmHg) were not significant, as indicated by CIs overlapping a value of 1.

**Table 4:** Cox proportional hazards regression models for systolic blood pressure with criminal offending.

	Hazard Ratio (95% CI)	
Quintiles for SBP in mmHg	Adjusted HRs^a^	Adjusted HRs^b^
**All criminal convictions**		
1^st^ (87–113)	**1.26 (1.04–1.52)**	1.17 (0.94–1.45)
2^nd^ (114–119)	1.04 (0.84–1.30)	1.12 (0.89–1.42)
3^rd^ (120–125)	0.84 (0.69–1.02)	**0.80 (0.65–0.99)**
4^th^ (126–133)	0.87 (0.71–1.06)	0.91 (0.73–1.12)
5^th^ (134–161)	1^c^	1^c^
**Violent convictions**		
1^st^ (87–113)	**2.55 (1.32–4.93)**	**2.52 (1.15–5.49)**
2^nd^ (114–119)	1.04 (0.43–2.50)	1.21 (0.46–3.22)
3^rd^ (120–125)	1.35 (0.68–2.67)	1.46 (0.67–3.19)
4^th^ (126–133)	1.03 (0.48–2.19)	1.28 (0.56–2.92)
5^th^ (134–161)	1^c^	1^c^
**Non-violent convictions**		
1^st^ (87–113)	1.20 (0.99–1.46)	1.10 (0.88–1.37)
2^nd^ (114–119)	1.06 (0.84–1.32)	1.13 (0.89–1.43)
3^rd^ (120–125)	**0.80 (0.66–0.98)**	**0.76 (0.61–0.94)**
4^th^ (126–133)	0.87 (0.71–1.06)	0.89 (0.72–1.10)
5^th^ (134–161)	1^c^	1^c^

Abbreviations: SBP (systolic blood pressure), mmHg (millimeter of mercury), CI (confidence interval).

^a^Adjusted for birth year

^b^Adjusted for birth year, physical energy capacity, height, and weight.

^c^Category of comparison.

In fully adjusted models, we found that female conscripts with the lowest SBPs (≤113 mmHg) compared to female conscripts with the highest SBPs (≥134 mmHg) had an increased risk of violent criminal convictions (HR = 2.52, 95% CI: 1.15, 5.49). In fully adjusted models we further found that female conscripts in the third SBP quintile (120–125 mmHg) compared to the female conscripts with the highest SBPs (≥134 mmHg) had a reduced risk of non-violent criminal convictions (HR = 0.76, 95% CI: 0.61, 0.94).

Despite few significant associations between SBP and any criminal convictions, violent criminal convictions and non-violent convictions, a test of all categories (i.e., SBP quintiles) being null simultaneously yielded a p-value < .05 for any criminal conviction, a p-value = 0.12 for violent criminal convictions, and a p-value < .05 for non-violent criminal convictions, indicating that SBP was associated with crime.

### Unintentional injuries

Table 5 displays the results from the Cox proportional hazards regression models for RHR and SBP with unintentional injuries. Unadjusted estimates are shown in S2 and S3 Tables of the Supporting Information. The fully adjusted models, analyses showed that female conscripts with the lowest RHRs (≤62 bpm) had an increased risk of unintentional injury (HR = 1.22, 95% CI: 1.08, 1.37) compared to female conscripts with the highest RHRs (≥83 bpm). For female conscripts in the second RHR quintile (63–69 bpm), the association between RHR and unintentional injury was not significant, as indicated by CIs overlapping a value of 1. However, female conscripts in the third quintile of RHR (70–75 bpm) had an increased risk of being unintentionally injured (HR = 1.13, 95% CI: 1.01, 1.27) relative to female conscripts with the highest RHRs (≥83 bpm). Similar results were found for female conscripts in the fourth quintile of RHR (76–82 bpm), who had an increased risk of being unintentionally injured (HR = 1.16, 95% CI: .03, 1.30) compared to female conscripts with the highest RHRs (≥83 bpm).

**Table 5:** Cox proportional hazards regression models for resting heart rate and systolic blood pressure with unintentional injuries.

Unintentional injury	Hazard Ratio (95% CI)	
Quintiles for RHR in bpm	Adjusted HRs^a^	Adjusted HRs^b^
1^st^ (38–62)	**1.20 (1.08–1.33)**	**1.22 (1.08–1.37)**
2^nd^ (63–69)	1.07 (0.96–1.18)	1.06 (0.94–1.19)
3^rd^ (70–75)	1.10 (0.99–1.21)	**1.13 (1.01–1.27)**
4^th^ (76–82)	**1.16 (1.05–1.28)**	**1.16 (1.03–1.30)**
5^th^ (83–145)	1^c^	1^c^
**Quintiles for SBP in mmHg**		
1^st^ (87–113)	0.96 (0.87, 1.06)	0.99 (0.89, 1.10)
2^nd^ (114–119)	0.91 (0.81, 1.01)	0.94 (0.83, 1.06)
3^rd^ (120–125)	0.92 (0.84, 1.00)	0.94 (0.85, 1.04)
4^th^ (126–133)	0.97 (0.89, 1.07)	0.97 (0.87, 1.07)
5^th^ (134–161)	1^c^	1^c^

Abbreviations: RHR (resting heart rate), SBP (systolic blood pressure), bpm (beats per minute), mmHg (millimeter of mercury), CI (confidence interval).

^a^Adjusted for birth year

^b^Adjusted for birth year, physical energy capacity, height, and weight.

^c^Category of comparison.

A test of all categories (i.e., RHR and SBP quintiles) being null simultaneously yielded a p-value < .05 for RHR and a p-value = 0.65 for SBP, indicating that RHR but not SBP was associated with unintentional injuries.

### Sensitivity analyses

Estimates for RHR were largely unaffected by restricting the analyses to include only female conscripts who had conscripted before age 21 (S4 Table). Estimates were further unaffected by stratifying the sample to include only those with the outcome occurring after conscription. However, given the reduced statistical power, the CIs were wider and overlapped a value of 1 (S5 Table).

Analyses comparing female conscripts to the female non-conscripts showed that female conscripts had higher childhood SES (*p* < .001) and a higher level of achieved education (*p* < .001; see S6 Table of the Supporting Information), but not a higher risk of being diagnosed with a psychiatric disorder (HR = 0.96, 95% CI: 0.92, 1.00). Female conscripts had a lower risk of being convicted of any crime (HR = 0.86, 95% CI: 0.81, 0.91), or of either a violent (HR = 0.68, 95% CI: 0.57, 0.81), or non-violent crime (HR = 0.86, 95% CI: 0.82, 0.91), but showed a higher risk of unintentional injury (HR = 1.41, 95% CI: 1.37, 1.45) relative to female non-conscripts (S7 Table of the Supporting Information).

## Discussion

Findings from the present study utilizing a sample of 12,499 female conscripts in Sweden showed that lower RHR measured at conscription was associated with an increased risk of any criminal offending. When subdividing the criminal offending outcome, a lower RHR was further associated with an increased risk of non-violent criminal offending. The corresponding association between lower RHR and violent crime was not statistically significant, likely due to insufficient statistical power (0.7% of the sample had a violent conviction). This is the first time the association between RHR and criminal offending has been demonstrated in a large-scale, all-female sample over a long-term follow-up period (i.e., up to 40 years). However, with crime as the outcome, results from tests of all RHR categories (i.e., quintiles) being null simultaneously was not significant (although SBP was). While we cannot rule out that this indicates that there is no association between RHR and crime, this too likely reflects insufficient statistical power. Either way, it is important to interpret findings from the present study with caution. In addition, results from the present study suggest that lower RHR was associated with an increased risk of unintentional injuries–an outcome that in prior research has been interpreted as potentially reflecting fearless and stimulation-seeking tendencies [9, 14].

Results from the present study not only corroborate earlier findings but also extend them theoretically as well as empirically [17–19]. The first study to investigate autonomic nervous system activity and its relation to antisocial behavior in females used a sample of 44 12–13-year-old girls who were screened for personality characteristics and disruptive behavior. Measures of heart rate discriminated between three behaviorally defined groups, confirming similar studies with boys [17]. Another study reported that a lower heart rate was found among aggressive children compared to non-aggressive children [19]. Children with lower heart rates were also more aggressive than children with higher heart rates. In this study, there was no evidence of an interaction with sex, suggesting similar results for boys and girls [19]. The most recent study to examine measures of heart rate in relation to antisocial behavior in females utilized a large sample of up to 2,256 women [18]. Interestingly, this study found lower RHR at age 18 to predict violent and non-violent crime among women, but lower RHR at age 11 or 15 did not predict violent and/or non-violent crime. For men, the association between lower RHR and violent and non-violent crime was robust across adolescence. Results from this study showed that the RHR was less stable across time for females as compared to males, something that may influence the results. Another possible explanation highlighted in this study was that lower RHR has been associated with persistent offending [11, 34], whereas women engage in this type of antisocial behavior to a lesser extent than men [18]. The earlier work with a focus on RHR and antisocial behavior in women has been limited by small and selected samples, a limitation that the present study sought to overcome by utilizing population-based data. Our study findings are interesting, in that they provide further indications of lower RHR as a potential predictor of criminal offending in women. Findings from the present study further suggest that higher RHR may operate in protective manner for crime among female conscripts, a possibility that has also been suggested in earlier work [35, 36].

Findings from the present study show that the association between RHR and criminal offending was evident in a sample of female conscripts who, according to sensitivity analyses, had a lower risk of criminal offending compared to female non-conscripts. Our finding that lower RHR was associated with an elevated risk of unintentional injuries among female conscripts is notable in light of prior evidence that lower RHR is also associated with a tendency to engage in extreme sports, such as skydiving [37], and with risky jobs such as bomb disposal work [12]. In contrast to the lower risk of criminal offending among female conscripts, the present study found that female conscripts had a higher risk of unintentional injuries compared to female non-conscripts. Perhaps the higher risk of unintentional injury and the lower risk of criminal offending in female conscripts reflects a fearless propensity [9] that is, on average, more prosocial than antisocial in nature given their lower rate of crime. Future research should explore whether there are ways to define subtypes of individuals with lower RHR and higher SES that can be used to explain when this physiological characteristic is associated with an increased risk of antisocial as opposed to prosocial outcomes.

Women in general have higher RHRs than men, beginning at an early age and continuing throughout life [15]. This has raised the question of whether lower RHR, and psychological attributes associated with it such as fearlessness, could provide a potential explanation for the greater involvement of men in crime [21]. Earlier work has demonstrated that lower RHR is a mediator in the association between sex and criminal offending, suggesting that lower RHR may in part explain the sex difference in crime rates [21]. Results from the present study suggest that lower RHR constitutes a potential risk factor for criminal offending also in women, but it may well be that it is a less important risk factor given the relatively higher RHR of women in general. Future research should therefore continue to explore the extent to which lower RHR can account for the salient sex difference in crime.

In contrast to women having a higher RHR compared to men, women tend to have lower blood pressure than men which is evident from puberty throughout adulthood up until menopause [38]. The suggested reasons for this difference in blood pressure between men and women has mainly been attributed to biological factors such as differences in hormones [39], especially sex hormones which relates to pregnancy and menopause [40], but also social factors such as stress given the potentially different lived experiences between men and women (i.e., gender inequality) [41]. While a lower SBP has been associated with a higher risk of criminal offending as well as reoffending in men [9, 11], the same distinct pattern was not observed for women. While the reason for this finding remain unclear, future studies should address potential sex differences in blood pressure in relation to criminal offending.

Associations between RHR and criminal offending, as well as unintentional injuries, remained largely unaffected by the adjustment for potentially important covariates, such as height, weight, and physical energy capacity. These findings indicate that RHR contributes uniquely to the prediction of these outcomes among female conscripts. Theoretically, lower RHR reflects lesser autonomic arousal, which may predispose to criminal behavior because it reflects a diminished capacity for fear [42]. In accordance with these theoretical presumptions, criminal offenders who exhibit lower autonomic arousal may be less able to learn from their experiences. For example, a lower level of fear may result in weakened aversive conditioning, which theoretically increases the risk for criminal offending [40]. Results from the present study corroborate earlier findings with males [9] in an all-female sample, showing not only that lower RHR is associated with an increased risk of criminal offending, but also of unintentional injuries. These findings in turn accord with the idea that low RHR reflects fearless/stimulation-seeking, as individuals who are lacking in fear may likely be more prone to engage in risky activities that give rise to injuries [14]. However, it will be important for future work to explore the aforementioned relations within the context of distinct dispositional traits (e.g., boldness and disinhibition) in order to further clarify the role fearlessness and stimulation seeking may play in the associations between 1) RHR and crime and 2) RHR and unintentional injuries in women.

In contrast to earlier findings in male samples where SBP has been demonstrated to be associated with criminal offending (see [9–11]), we could not confirm that lower SBP was related to criminal offending in female conscripts, apart from the finding of higher risk for violent offending among women in the lowest SBP quintile relative to those in the highest. However, the largely null results for SBP could to some extent be reflecting limited statistical power, given the wide CIs around the estimates for this measure. Investigation of SBP as a predictor of criminal offending is still in its early stages and more research is needed before firm conclusions can be drawn.

### Limitations of the study

The findings from the present study should be considered in light of some limitations. RHR was measured using an arm-cuff monitor, following standard procedure in clinical practice in Sweden [26]. However, this procedure may be less sensitive than a laboratory assessment in which electrodes are attached to the limbs or torso to record RHR data. Other factors that can influence measurement of cardiovascular activity, such as room temperature and time of day, were not available from the Swedish Military Conscription Register and thus could not be controlled for. Further, it is possible that a portion of the unintentional injuries in the present study are not associated with fearless or risk-taking behavior, but rather other reasons such as general reckless behavior. We were not able to distinguish between the behavioral circumstances for the unintentional injuries which is why results should be interpreted with caution. We did not include more covariates because we aimed at describing the associations between autonomic arousal and the outcomes, rather than testing causality. Testing for causality would require use of for example genetically sensitive designs, something that would be desirable for future studies to address.

Perhaps the most important limitation of the present study is the selected nature of the sample. Even though it represents a relatively large sample, comprising over 12,000 women, female conscripts are not likely to be representative of the overall female population. While representativeness is important for generalizability, it could be argued that it is not always needed to make valid scientific inferences [43]. Having said that, we can rule out that observed associations are influenced by selection bias [44]. To assess for potential differences between female conscripts and female non-conscripts, we compared the two groups of women on several key descriptive variables using sensitivity analyses. Findings showed that female conscripts and female non-conscripts differed on certain factors, and these differences should be considered in interpreting the study results. For example, female conscripts were less crime-prone compared to female non-conscripts, which could have attenuated the association between RHR/SBP and criminal offending in the present study.

Another further limitation of the present study is statistical power, which did not allow us to adjust for childhood SES as has been done in previous studies (see [9–11]). Despite the relatively large size of our study sample, CIs around some of our reported effects were wide, providing more reason for caution in interpreting and drawing conclusions from the present findings. Additionally, as in any correlational study, there are various uncontrolled factors that could have influenced our results. For example, we relied on official criminal offenses recorded in the National Crime Register, which likely reflect more visible and severe infractions committed by members of society. Further research should undertake to replicate the current findings using self-reported crime data collected directly from members of the general population. It is always an issue in observational study designs that there may be other confounding factors that we were unable to control for. We relied on official crime data from the National Crime Register, which is likely to reflect the more severe cases at large. Thus, future research should replicate these findings using self-reported crime data from the general population.

Notwithstanding these limitations, findings from the present study demonstrate a negative association between RHR and criminal offending in the largest female sample investigated to date, even after adjusting for potentially important covariates. We also found evidence of an association between lower SBP and violent criminal convictions specifically. While these results warrant replication, findings indicate that reduced autonomic arousal, as indexed by lower RHR in particular, may have the potential to serve as a predictor of criminal offending, not only in men as suggested by previous studies, but also in women.

## Supporting information

S1 Table Descriptive information for female non-conscripts. (DOCX)

S2 Table Unadjusted Cox proportional hazards regression model for resting heart rate with criminal offending and unintentional injury. (DOCX)

S3 Table Unadjusted Cox proportional hazards regression models for systolic blood pressure with criminal offending and unintentional injury. (DOCX)

S4 Table Cox proportional hazards regression models for resting heart rate with criminal offending and unintentional injury among female conscripts who conscripted before age 21. (DOCX)

S5 Table Cox proportional hazard regression models for resting heart rate with criminal offending and unintentional injury among female conscripts who were conscripted before experiencing the outcome. (DOCX)

S6 Table Comparing female conscripts to female non-conscripts on demographic factors using Mann-Whitney U test. (DOCX)

S7 Table Comparing female conscripts to female non-conscripts on psychiatric disorders and outcome variables using Cox proportional hazards regression. (DOCX)

S1 Fig Kaplan-Meier survival curves for RHR with criminal convictions and unintentional injuries. (TIF)

S2 Fig Kaplan-Meier survival curves for SBP with criminal convictions and unintentional injuries. (TIF)
