# Supplementary material for: Lower autonomic arousal as a risk factor for criminal offending and unintentional injuries among female conscripts
Source: PLoS One. 2024 Mar 27;19(3):e0297639. doi: 10.1371/journal.pone.0297639 (PMC10971584; doi:10.1371/journal.pone.0297639)
Supplement: S1 Table — (DOCX) [file pone.0297639.s001.docx]

**S1 Table. Descriptive Information for Female Non-Conscripts.**

|  | **No. (%) with data** | **M (SD)** |
| --- | --- | --- |
| **Total** | 1,714,152 (100.0) |  |
| **Variables** |  |  |
| Age at first criminal conviction, y | 178,149 (10.4) | 21.9 (7.9) |
| Age at first violent criminal conviction, y | 20,322 (1.2) | 24.0 (9.1) |
| Age at first non-violent criminal conviction, y | 169,945 (9.9) | 21.9 (7.9) |
| Age at first unintentional injury, y | 444,689 (25.9) | 31.3 (10.8) |
|  |  |  |
| **Any psychiatric disorder** |  |  |
| Yes | 301,712 (17.6) |  |
| No | 1,412,440 (82.4) |  |
| **Childhood SES** |  |  |
| Low | 601,938 (35.1) |  |
| Medium | 528,182 (30.8) |  |
| High | 300,516 (17.5) |  |
| Missing | 283,516 (16.5) |  |
| **Highest achieved education** |  |  |
| Low | 412,478 (24.0) |  |
| Medium | 796,311 (46.5) |  |
| High | 487,884 (28.5) |  |
| Missing | 17,479 (1.0) |  |

Abbreviations: y (years), SES (socioeconomic status).
