# Supplementary material for: Lower autonomic arousal as a risk factor for criminal offending and unintentional injuries among female conscripts
Source: PLoS One. 2024 Mar 27;19(3):e0297639. doi: 10.1371/journal.pone.0297639 (PMC10971584; doi:10.1371/journal.pone.0297639)
Supplement: S2 Table — (DOCX) [file pone.0297639.s002.docx]

**S2 Table. Unadjusted Cox Proportional Hazards Regression Model for Resting Heart Rate with Criminal Offending and Unintentional Injuries.**

|  | **Hazard Ratio (95% CI)** |
| --- | --- |
| **Quintiles for RHR in bpm** | **Unadjusted HRs** |
| **All criminal convictions** |  |
| 1^st^ (38-62) | **1.28 (1.02-1.60)** |
| 2^nd^ (63-69) | 1.23 (0.99-1.54) |
| 3^rd^ (70-75) | **1.25 (1.00-1.57)** |
| 4^th^ (76-82) | 1.15 (0.91-1.44) |
| 5^th^ (83-145) | 1^a^ |
| **Violent convictions** |  |
| 1^st^ (38-62) | 1.32 (0.65-2.66) |
| 2^nd^ (63-69) | 1.34 (0.67-2.68) |
| 3^rd^ (70-75) | 0.96 (0.45-2.07) |
| 4^th^ (76-82) | 1.12 (0.54-2.33) |
| 5^th^ (83-145) | 1^a^ |
| **Non-violent convictions** |  |
| 1^st^ (38-62) | **1.30 (1.03-1.65)** |
| 2^nd^ (63-69) | **1.27 (1.01-1.60)** |
| 3^rd^ (70-75) | **1.33 (1.06-1.68)** |
| 4^th^ (76-82) | 1.20 (0.95-1.52) |
| 5^th^ (83-145) | 1^a^ |
| **Unintentional injuries** |  |
| 1^st^ (38-62) | **1.38 (1.25-1.53)** |
| 2^nd^ (63-69) | **1.18 (1.07-1.30)** |
| 3^rd^ (70-75) | **1.19 (1.08-1.32)** |
| 4^th^ (76-82) | **1.20 (1.09-1.33)** |
| 5^th^ (83-145) | 1^a^ |

Abbreviations: RHR (resting heart rate), bpm (beats per minute).

^a^Category of comparison.
