# Supplementary material for: Lower autonomic arousal as a risk factor for criminal offending and unintentional injuries among female conscripts
Source: PLoS One. 2024 Mar 27;19(3):e0297639. doi: 10.1371/journal.pone.0297639 (PMC10971584; doi:10.1371/journal.pone.0297639)
Supplement: S3 Table — (DOCX) [file pone.0297639.s003.docx]

**Table S3. Unadjusted Cox Proportional Hazards Regression Models for Systolic Blood Pressure with Criminal Offending and Unintentional Injury.**

|  | **Hazard Ratio (95% CI)** |
| --- | --- |
| **Quintiles for SBP in mmHg** | **Unadjusted HRs** |
| **All criminal convictions** |  |
| 1^st^ (87-113) | **1.22 (1.01-1.48)** |
| 2^nd^ (114-119) | 1.04 (0.84-1.30) |
| 3^rd^ (120-125) | 0.83 (0.69-1.01) |
| 4^th^ (126-133) | 0.87 (0.71-1.06) |
| 5^th^ (134-161) | 1^c^ |
| **Violent convictions** |  |
| 1^st^ (87-113) | **2.46 (1.27-4.75)** |
| 2^nd^ (114-119) | 1.01 (0.42-2.45) |
| 3^rd^ (120-125) | 1.34 (0.68-2.66) |
| 4^th^ (126-133) | 1.02 (0.48-2.18) |
| 5^th^ (134-161) | 1^c^ |
| **Non-violent convictions** |  |
| 1^st^ (87-113) | 1.16 (0.96-1.41) |
| 2^nd^ (114-119) | 1.05 (0.84-1.31) |
| 3^rd^ (120-125) | **0.79 (0.65-0.96)** |
| 4^th^ (126-133) | 0.87 (0.71-1.06) |
| 5^th^ (134-161) | 1^c^ |
| **Unintentional injuries** |  |
| 1^st^ (87-113) | **1.13 (1.03-1.24)** |
| 2^nd^ (114-119) | 0.95 (0.85-1.06) |
| 3^rd^ (120-125) | 1.01 (0.92-1.10) |
| 4^th^ (126-133) | 0.99 (0.90-1.08) |
| 5^th^ (134-161) | 1^c^ |

Abbreviations: SBP (systolic blood pressure), mmHg (millimeter of mercury), CI (confidence interval). ^a^Adjusted for birth year; ^b^Adjusted for birth year, physical energy capacity, height, and weight. ^c^Category of comparison.
