# Supplementary material for: Lower autonomic arousal as a risk factor for criminal offending and unintentional injuries among female conscripts
Source: PLoS One. 2024 Mar 27;19(3):e0297639. doi: 10.1371/journal.pone.0297639 (PMC10971584; doi:10.1371/journal.pone.0297639)
Supplement: S4 Table — (DOCX) [file pone.0297639.s004.docx]

**Table S4. Cox Proportional Hazards Regression Models for Resting Heart Rate with Criminal Offending among Female Conscripts who Conscripted Before Age 21.**

|  | **Hazard Ratio (95% CI)** |
| --- | --- |
| **Quintiles for RHR in bpm** | **Adjusted HRs^a^** |
| **All criminal convictions** |  |
| 1^st^ (38-62) | **1.33 (1.01-1.77)** |
| 2^nd^ (63-69) | **1.36 (1.03-1.79)** |
| 3^rd^ (70-75) | 1.29 (0.98-1.70) |
| 4^th^ (76-82) | 1.13 (0.85-1.50) |
| 5^th^ (83-145) | 1^b^ |
| **Violent convictions** |  |
| 1^st^ (38-62) | 1.44 (0.59-3.53) |
| 2^nd^ (63-69) | 1.89 (0.84-4.26) |
| 3^rd^ (70-75) | 1.03 (0.41-3.64) |
| 4^th^ (76-82) | 0.78 (0.28-2.18) |
| 5^th^ (83-145) | 1^b^ |
| **Non-violent convictions** |  |
| 1^st^ (38-62) | **1.35 (1.01-1.81)** |
| 2^nd^ (63-69) | **1.37 (1.03-1.83)** |
| 3^rd^ (70-75) | **1.37 (1.03-1.81)** |
| 4^th^ (76-82) | 1.19 (0.89-1.59) |
| 5^th^ (83-145) | 1^b^ |
| **Unintentional injury** |  |
| 1^st^ (38-62) | **1.19 (1.05-1.35)** |
| 2^nd^ (63-69) | 1.02 (0.90-1.16) |
| 3^rd^ (70-75) | 1.10 (0.97-1.24) |
| 4^th^ (76-82) | 1.12 (0.99-1.27) |
| 5^th^ (83-145) | 1^b^ |

Abbreviations: RHR (resting heart rate), bpm (beats per minute), CI (confidence interval). ^a^Adjusted for birth year, physical energy capacity, height, and weight. ^b^Category of reference.
