# Supplementary material for: Lower autonomic arousal as a risk factor for criminal offending and unintentional injuries among female conscripts
Source: PLoS One. 2024 Mar 27;19(3):e0297639. doi: 10.1371/journal.pone.0297639 (PMC10971584; doi:10.1371/journal.pone.0297639)
Supplement: S5 Table — (DOCX) [file pone.0297639.s005.docx]

**Table S5. Cox Proportional Hazard Regression Models for Resting Heart Rate with Criminal Offending and Unintentional Injury among Female Conscripts who were Conscripted before Experiencing the Outcome.**

|  | **Hazard Ratio (95% CI)** |
| --- | --- |
| **Quintiles for RHR in bpm** | **Adjusted HRs^a^** |
| **All criminal convictions** |  |
| 1^st^ (38-62) | 1.28 (0.85-1.92) |
| 2^nd^ (63-69) | 1.28 (0.87-1.89) |
| 3^rd^ (70-75) | 1.30 (0.88-1.92) |
| 4^th^ (76-82) | 0.96 (0.64-1.46) |
| 5^th^ (83-145) | 1^b^ |
| **Non-violent convictions** |  |
| 1^st^ (38-62) | 1.35 (0.88-2.07) |
| 2^nd^ (63-69) | 1.30 (0.86-1.98) |
| 3^rd^ (70-75) | 1.47 (0.98-2.20) |
| 4^th^ (76-82) | 1.08 (0.70-1.65) |
| 5^th^ (83-145) | 1^b^ |
| **Unintentional injury** |  |
| 1^st^ (38-62) | **1.21 (1.07-1.36)** |
| 2^nd^ (63-69) | 1.06 (0.94-1.19) |
| 3^rd^ (70-75) | 1.12 (0.99-1.26) |
| 4^th^ (76-82) | **1.15 (1.02-1.30)** |
| 5^th^ (83-145) | 1^b^ |

Abbreviations: RHR (resting heart rate), bpm (beats per minute), CI (confidence interval). ^a^Adjusted for birth year, physical energy capacity, height, and weight. ^b^Category of reference.
