# Supplementary material for: Lower autonomic arousal as a risk factor for criminal offending and unintentional injuries among female conscripts
Source: PLoS One. 2024 Mar 27;19(3):e0297639. doi: 10.1371/journal.pone.0297639 (PMC10971584; doi:10.1371/journal.pone.0297639)
Supplement: S6 Table — (DOCX) [file pone.0297639.s006.docx]

**Table S6. Comparing Female Conscripts to Female Non-Conscripts on Demographic Factors Using Mann-Whitney U Test.**

|  | **Female non-conscripts** | | | **Female conscripts** | | |  |  |
| --- | --- | --- | --- | --- | --- | --- | --- | --- |
|  | *M* | *Md* | *Range* | *M* | *Md* | *Range* | *W* | *p* |
| **Childhood SES** | 1.8 | 2.0 | 1-3 | 1.9 | 2.0 | 1-3 | 7908 | <.001 |
| **Highest achieved education** | 2.0 | 2.0 | 1-3 | 2.3 | 2.0 | 1-3 | 1047 | <.001 |
