# Supplementary material for: Lower autonomic arousal as a risk factor for criminal offending and unintentional injuries among female conscripts
Source: PLoS One. 2024 Mar 27;19(3):e0297639. doi: 10.1371/journal.pone.0297639 (PMC10971584; doi:10.1371/journal.pone.0297639)
Supplement: S7 Table — (DOCX) [file pone.0297639.s007.docx]

**Table S7. Comparing Female Conscripts to Female Non-Conscripts on Psychiatric Disorders and Outcome Variables Using Cox Proportional Hazards Regression.**

|  | **Female non-conscripts**  **(n=1,714,152)** | **Female conscripts**  **(n=12,499)** | **HR (95% CI)^a^** |
| --- | --- | --- | --- |
|  | *n (%)* | *n (%)* |  |
| **Any psychiatric disorder** | 301,712 (17.6) | 1,947 (15.6) | 0.96 (0.92, 1.00) |
| **Any criminal conviction** | 178,149 (10.4) | 952 (7.6) | **0.86 (0.81, 0.91)** |
| **Violent criminal conviction** | 20,322 (1.2) | 84 (0.7) | **0.68 (0.57, 0.81)** |
| **Non-violent criminal conviction** | 169,945 (9.9) | 910 (7.3) | **0.86 (0.82, 0.91)** |
| **Unintentional injuries** | 444,689 (25.9) | 4,263 (34.1) | **1.41 (1.37, 1.45)** |

Abbreviations: HR (Hazard Ratio), CI (confidence interval)

^a^Adjusted for birth year.
