# Supplementary figures and images for: Lower autonomic arousal as a risk factor for criminal offending and unintentional injuries among female conscripts
Source: PLoS One. 2024 Mar 27;19(3):e0297639. doi: 10.1371/journal.pone.0297639 (PMC10971584; doi:10.1371/journal.pone.0297639)

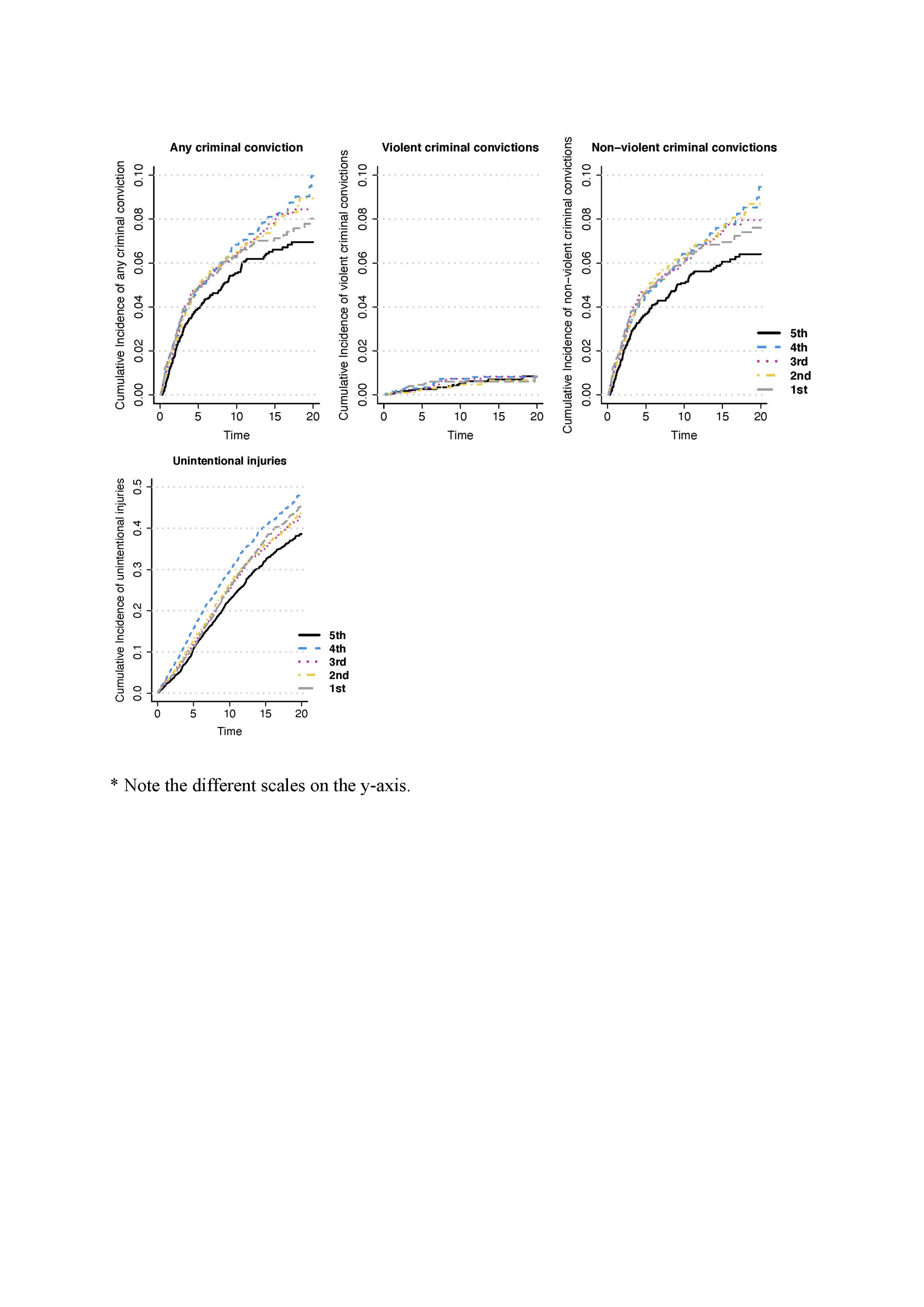

Supplement: S1 Fig — (TIF) [file pone.0297639.s008.tif]

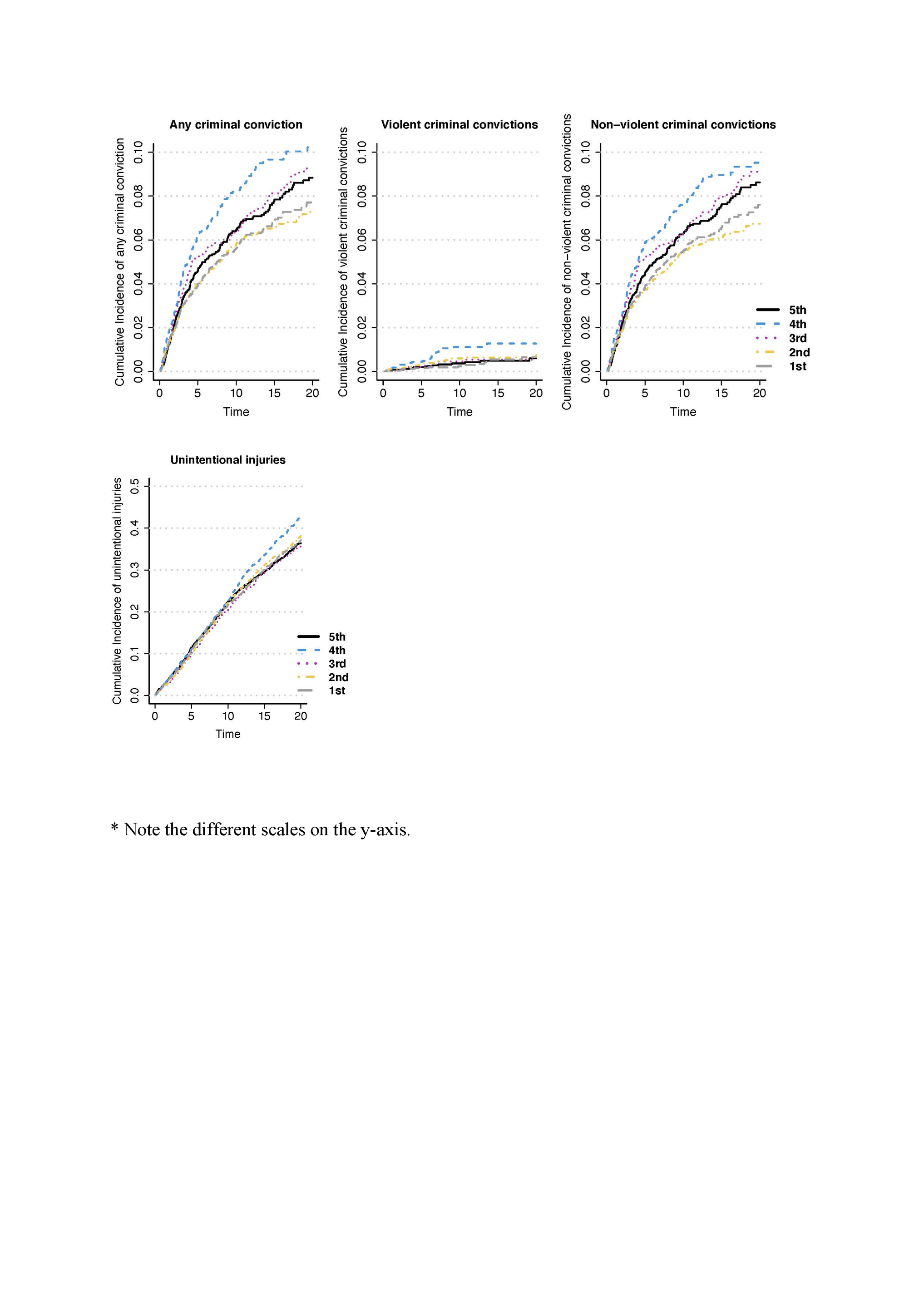

Supplement: S2 Fig — (TIF) [file pone.0297639.s009.tif]
